# Supplementary material for: Are carbon emissions trading and green financial instruments synergistic? -Comprehensive quantitative research based on content analysis
Source: PLoS One. 2024 Mar 7;19(3):e0298601. doi: 10.1371/journal.pone.0298601 (PMC10919601; doi:10.1371/journal.pone.0298601)
Supplement: S1 Data — (ZIP) [file pone.0298601.s001.zip › Data/地级市Z_F工作报告.zip!/║■─╧/╒┼╝╥╜τ╩╨/└┤╫╘╣1⁄2╓┌║┼í╛┬φ┐╦╩2╛▌═°í┐_2015.docx]

各位代表：

现在，我代表市人民政府向大会作工作报告，请予审议，并请各位政协委员和其他列席人士提出意见。

一、2014年工作回顾

2014年，是我市经济社会发展迎难而上、稳步前行的一年。面对“三期叠加”效应凸显、经济下行压力加大等多重困难，全市上下以深入开展党的群众路线教育实践活动为契机，认真贯彻落实党中央、国务院重大决策部署，在省委、省政府和市委正确领导下，在市人大、市政协的监督支持下，紧紧围绕建设国内外知名旅游胜地和全面建成小康社会目标任务，全面实施“提质张家界，打造升级版”战略及“1656行动计划”，提振信心，攻坚克难，锐意奋进，致力稳增长、促改革、调结构、惠民生，保持了经济社会稳中有进、总体向好的发展态势。

（一）全力以赴稳定经济增长。一年来，根据国内外经济发展新形势新变化，我们保持定力，精准发力，千方百计稳增长、促发展。一是大力推动旅游升温。积极把握旅游市场“理性回归”大势，狠抓活动、政策、事件、媒体、客源地“五大营销”,加快品牌、项目、环境、交通、体制机制“五大建设”，努力实现旅游经济较快增长。二是突出抓好投资拉动。充分发挥投资对经济发展的关键作用，力争国投省投资金，创新扩大直接融资；坚持把项目建设摆在更加重要的位置，扎实开展“项目建设突破年”活动，全力实施基础设施、产业发展、民生改善项目建设，切实把稳增长落实到项目工程上、把促发展体现在投资落地上。三是积极引导房地产市场稳健发展。针对房地产市场持续下滑局面，按照“控总量、去存量、保质量”的原则，适时制定出台和实施刺激房地产市场“张十条”等一系列政策举措，增强市场信心，激发市场活力。四是不断加大金融支持力度。按照“扩需求、稳增量、抓改革、控风险”的基本思路，科学统筹金融改革创新和经济社会发展，拓展政银企合作平台，加强项目信贷协调，有效扩大信贷投放，全市金融机构新增贷款突破50亿元，同比增长17.2%。五是扎实推进招商引资。实施重大项目招商责任制，突出重点领域、重点产业、重点企业、重点项目招商，充分利用“沪洽周”、“珠洽会”等招商平台，成功引进了大连万达、河北卓达、深圳金赛银等一批战略投资者，共签约重大项目32个，投资总额达到456亿元。六是进一步强化建设用地保障。及时开展土地利用总体规划修编，进一步拓展中心城区建设用地空间，有力保障了项目落地建设。全年获批建设用地439.5公顷，实际供应用地595.07公顷，同比分别增长21.13%和37.63%。

通过扎实努力，全市实现了经济平稳健康发展。预计（下同）全市生产总值突破400亿元大关，达到410亿元，同比增长10.1%，其中一、二、三次产业同比分别增长4.7%、7.7%、12.2%。实现游客接待总量3884万人次、过夜游客1644万人次、旅游总收入246亿元，同比分别增长12%、16%、16%；规模工业增加值同比增长8.7%；社会消费品零售总额138.9亿元，同比增长12%；外贸进出口总额8500万美元，同比增长85.5%；固定资产投资253亿元，同比增长20%；实际利用外资8041万美元、内资40.7亿元，同比分别增长20%和10%；财政总收入40.6亿元，同比增长12%；城乡居民人均可支配收入分别达到18238元和6802元，同比分别增长10%和12%；城镇登记失业率和居民消费价格涨幅分别控制在3.48%和2%。对照年初预期目标，生产总值、旅游接待总量、过夜游客人数、旅游总收入、农业增加值、实际利用外资内资、外贸进出口总额等八项指标超额完成；固定资产投资、财政总收入、农村居民人均可支配收入、城镇登记失业率、居民消费价格涨幅等五项指标达到预期。受经济下行压力加大、企业生产经营困难等全局性不利因素影响，规模工业增加值、城镇居民人均可支配收入、社会消费品零售总额等三项指标未达到预期。

（二）旅游规模和效益再创历史新高。坚持一手抓转型，一手抓营销，调结构，扩总量，旅游经济主要指标全面超额完成年度任务，再创历史最高水平。旅游营销精准发力。全面提高营销的针对性和实效性，强化市场营销和景区门票奖励等一系列促销政策，成功举办了第三届世界翼装飞行锦标赛、中韩高尔夫名人挑战赛和全国网络大咖户外挑战赛等一系列营销活动，智慧旅游官网上线运行，媒体营销领域不断拓展，张家界版“小苹果”视频和“心花路放”电影等事件营销引起广泛关注。旅游大交通组织更加有效。开通了张家界至釜山国际定期航班，新增了张家界至长沙奥凯空中快线，荷花国际机场起降航班同比增长13.24%，其中出入境航班同比增长45.2%。开行了张家界至济南、深圳、太原旅游列车，张家界火车站停靠旅游专列同比增长了一倍。旅游服务功能不断完善。中心城区南门口、仙人溪等特色街区建设项目启动实施，武陵源溪布街提质升级加快实施。开发推出12条户外精品旅游线路，成功引进5家户外企业落地张家界。多层次、多类型的旅游酒店蓬勃发展，以高星级酒店为龙头、经济型酒店为主体、特色客栈为补充的宾馆酒店业体系正在形成。游客中心体系、旅游交通标识系统等旅游公共服务建设扎实推进，“安导通”项目正式运行，自驾游、自助游服务体系逐步完善。旅游市场环境进一步优化。目的地与客源地旅游联合协作执法机制初步形成，旅游服务质量监管和市场秩序监管体系初步建立，“平安满意在张家界”专项活动不断深化，世界地质公园顺利通过中期评估验收，旅游品牌美誉度和吸引力进一步提升。

（三）产业建设步伐明显加快。坚持“产业兴市、产业强市”路径，实施“调一进二提三”方略，产业发展总量、质量和效益同步提升。农业结构调整持续深化。依靠科技，主攻单产，粮食生产水平稳步提升。着力实施农业产业提质升级“523行动计划”，新增商品蔬菜基地4.2万亩、大鲵29.6万尾，同比分别增长29.4%、45.3%。扶持发展茶叶1.42万亩、油茶6万亩，同比分别增长21.8%、30%。农业新型经营主体快速成长，新发展市级以上农业产业化龙头企业35家，新增农民专业合作社100家，新扩农村土地承包经营权流转面积2.8万亩。农业安全发展保障能力增强，防汛抗旱、森林防火、重大动植物疫病有效防控，农业保险种类和覆盖面逐步扩展。工业经济建设力度加大。制定出台工业产业提质升级“135行动计划”，力促工业经济加快建设、良性发展。全市完成工业固定资产投资54.83亿元、技改投资40.46亿元，同比分别增长25%。承接产业转移项目17个，其中9个项目已经或即将建成投产。实现高新技术产业增加值5亿元，同比增长36.2%。完成工业园区基础设施建设投资4.9亿元，同比增长37%；新增入园企业18家，园区规模工业增加值占全市规模工业增加值的比重达到27%。现代服务业稳步发展。启动全市商贸流通产业发展规划编制，完成城市商业网点规划修订。城乡商贸流通基础设施建设不断加强，中商广场、家乐福、图书城等一批大型商业综合体建设加快推进，11家农贸市场标准化改造基本完成，粮食“危仓老库”改造工程全面实施。服务业市场主体快速发展，新登记商贸、信息、文体、科技服务类企业同比分别增长203.3%、244.4%、87.5%和71.4%。供销云商电子商务平台即将投入营运。

（四）重点工程建设取得重要进展。坚持基础先行、项目优先，相继实施重点工程项目76个，完成投资90亿元，同比增长50%。重大基础设施项目建设取得历史性突破。黔张常高铁破土动工，开启了张家界高铁时代，全市人民翘首多年的期盼终于得以实现。张桑高速公路开工兴建，正在改写贺龙元帅故里、革命老区桑植“无高速”的历史。荷花国际机场新航站楼建设顺利实施，作为湖南和武陵山片区对外开放的窗口形象将大为改观。岩泊渡、东洋渡大桥已建成通车，双门岛大桥、旅游航道和绕城西线、邢大、刘洪、官瑞等一批干线公路加快推进。通信网络、城乡电网、水利建设工程扎实推进，全面完成年度目标任务。重大产业发展项目相继建成运行。碧桂园凤凰酒店、禾田居国际度假酒店、华天酒店正式营业，盛美达休闲酒店二期和远大蓝色港湾酒店群一期主体工程完成，华新水泥技改工程竣工试运行，九城汽车物流城6家4S店营业，桑植、慈利页岩气勘探稳步推进。重大社会发展项目建设进程加快。贺龙体育中心主体工程完工，市博物馆地质馆开始布展，张家界文化广场即将建成，市图书馆开工兴建，张家界航院整体搬迁和市人民医院扩建工程加快实施，市技工学校搬迁一期工程、市二人民医院住院楼建成使用，张家界一中搬迁工程正式启动。

（五）城市建设管理水平大幅提升。坚持“在建设中管理，在管理中建设”，全面打造旅游城市良好形象。城市基础设施建设加快，运行功能不断增强。围绕“拓展新城两区、提质澧水两带”，大力实施中心城区“四大工程”建设。澧兰西路、澧兰东路和观音大桥南端匝道建成通车，回龙路平战结合项目主体工程完工，澧兰中路、澧兰桥、天问路、木龙滩路和“百巷改造”工程全面启动，沙堤大道建设前期工作扎实推进，澧水两岸截污管道整治工程顺利完成，东门溪雨污分流整治工程全面开工，澄潭二水厂提质工程完工，供水、供电、供气、通讯等市政配套设施进一步完善。城市管理实现突破，市容市貌焕然一新。强力实施“街道大清洗、垃圾大清运、渣土大整治、绿化大补植、停车大规范、烟花爆竹大整顿、市政设施大维护、违法建设大管控”等八大专项整治行动，主次干道实现常态化冲洗，建筑渣土及扬尘实现全方位规范化管理，清除卫生死角和城乡结合部积存垃圾20.95万吨，完成绿化补植3.5万平方米，投入5000 万元实施了市政设施全面维修。下大气力规范城区车辆行驶和停靠，基本实现了城区禁止烟花爆竹燃放和垃圾焚烧，基本杜绝了中心城市新的违法建设。城市环境卫生水平全面提升，被中国城市竞争力研究会评为“2014·中国十佳最干净城市”。城乡规划体系不断完善，法定图则管理有效加强。完成了城市总体规划实施评估和局部修改调整，编制了沙堤片区和武陵源城区控制性详细规划，启动了城市近期建设规划编制和市城区航拍地形图全面更新，完成了南门口、仙人溪特色街区概念性规划和澧水风貌带景观设计。乡镇规划全面完成，村镇规划进一步加强，镇（乡）域村镇布局规划和村庄规划覆盖率已分别达到67%和30%。

（六）民生改善和社会建设全面加强。坚持民生为本，加大民生投入，努力让老百姓在改革发展中得到更多实惠。民生实事全面落实。顺应人民群众期盼，认真办好了省政府安排的27件实事和市政府承诺的4件实事。新增城镇就业11092人，建成 1744套城市公租房和100所农村幸福院，改造城市棚户区民房2964 户、农村危房5430户，实施农村通畅工程290公里，解决了11.8万农村人口饮水安全问题。社会保障水平明显提高。新增“五大保险”参保2.18万人，社保基金征缴突破10亿元，达到10.7亿元。企业退休人员基本养老、城乡居民低保、农村五保户供养标准和新农合、城镇居民医保报销补偿率进一步提高，财政资金用于社保的支出同比增长9.36%。社会事业不断进步。一年来，教育、文体、科技、卫生事业加快发展。建成义务教育合格学校27所、农村公办幼儿园13所，添置农村寄宿制学校床位10668个。有效缓解中心城区中小学“大班额”问题，启动中心城区13所中小学校新建和扩容提质。城乡文化设施建设纵深推进，科技创新取得新成果，卫生和计生惠民政策全面落实，人口自然增长率控制在5.77‰。扶贫攻坚和新农村建设扎实推进。强化责任机制，大力实施扶贫攻坚“四基工程”，产业开发和资金扶持更加精准，实现了贫困人口减少年度目标。统筹全面小康和新农村示范建设，加大涉农资金和项目整合力度，不断推进投入机制创新，农村面貌全面改善。生态环境保护切实加强。国家主体功能区建设试点示范、首批生态文明先行示范区、生态文明示范工程试点市和全省首批低碳景区试点相继启动，大气、水、噪声等环境整治“六大专项行动”和“两型社会”建设取得实效。全市新增人工造林8.5万亩，创建市级生态村185个、省级生态乡镇3个，武陵源区跨入省级生态区行列。节能降耗完成省定目标任务，空气质量优良率保持全省领先水平。社会大局和谐稳定。落实安全生产责任，强化安全生产措施，安全生产形势总体平稳，重大安全生产事故得到有效遏制。食品药品、农产品“放心工程”深入推进，质量技术监管全面加强。信访维稳和反恐应急处突机制进一步完善，信访工作进入全省先进行列。大力深化“平安张家界”、“法治张家界”创建，不断强化社会治安综合治理和打击整治，禁毒、消防、交通管理均取得好成绩，社会治安状况民调排名全省第一。国防教育、民兵预备役建设和“双拥”工作进一步加强。桑植县白族乡“逢十庆典”活动取得圆满成功。

（七）各项改革统筹推进。一年来，全市上下认真落实中央和省委、省政府全面深化改革安排部署，统筹推进了行政体制、旅游管理、经济体制、社会事业等各项改革。政府机构改革启动实施，市级政府机构改革方案正式报批，减少处级机构15个，撤销议事协调机构292个。市县食品药品安全监管体制改革全面启动。完成新一轮行政审批制度改革，取消和下放审批事项167项，发布173项市级行政审批项目清单。事业单位改革分步实施，基本完成市直307个事业单位分类工作，为进一步深化事业单位改革奠定了基础。城管体制改革全面推进，按照“综合执法、重心下移、厘清责任、提高效能”的原则，调整理顺市、区城市管理体制，相对集中城市管理领域行政处罚权，积极探索城市园林养护、道路保洁、设施维护市场化运作。景区体制改革取得实质性进展，武陵源核心景区“政事分开、政企分开、事企分开、局处合一”改革方案启动实施，天门山先导区成功申报省级文化旅游产业园区试点。投融资体制改革取得新突破，实现直接融资17.37亿元，同比增长53.9%；金鲵生物等4家企业实现场外交易市场挂牌；非经营性政府投资项目代建制试点正式启动。工商登记制度改革成效明显，新登记企业和注册资本同比分别增长73.45%和224%。财税金融体制改革步伐加快，“营改增”试点总体平稳，服务业减税幅度超过30%。慈利、桑植农村信用联社股份制改革正式启动。教育改革力度加大，中心城区“幼升小”和“小升初”实现免试就近入学，探索推行了中小学校集团化办学模式。医药卫生体制改革深入推进，市人民医院、市二人民医院、市妇幼保健院“三院合一”启动实施，全市基本药物制度覆盖率达到85.75%。国企改革及其他改革稳步推进。

（八）政府自身建设成效突出。深入开展党的群众路线教育实践活动，狠抓政府自身建设，着力打造人民满意政府。政府机关工作作风明显好转。“四风”突出问题得到有效整治，充分发挥财政、审计、监察、编制等部门职能作用，共清退公车109辆、“吃空饷”人员80人、办公用房30137平方米，减少出境公务活动18批次，压缩“三公”经费1.43亿元，查办案件948起、处理相关责任人员700多名，制定出台了21个改进作风制度性规定。群众呼声得到及时回应，人民群众通过电视问政反映的涉农收费、抗旱应急水源建设、城镇保障房分配等突出问题全面整改落实，通过政府“12345”服务热线等渠道反映的各类实际困难和问题及时交办、跟踪督查、限时回应。群众对教育实践活动总体评价满意率达100%、对解决“四风”问题评价满意率达98.5%，全市绩效评估公众满意度评价位居全省前列。民主法治和依法行政全面推进。全市上下民主法治意识进一步增强，运用法治思维和法治方式推进改革发展稳定的能力进一步提高。切实贯彻执行依法行政“一规划两规定六办法”，认真落实重大行政决策法定程序；积极推行行政裁量权基准，审定发布一批行政执法指导案例，不断规范行政执法行为。着力强化政务服务中心运行，扎实推行网上审批、电子监察，有效提高行政服务水平。坚持定期向人大、政协报告和通报工作制度，自觉接受人大及其常委会法律监督和工作监督、政协民主监督以及社会各界监督，人大代表、政协委员对建议、提案办理满意率分别达到99.32%和99.34%。

一年来，全市各级各部门各单位履职尽责，积极作为，统计、民族、宗教、残联、老龄、外事、侨务、档案、史志、气象、移民、粮食、人防、对台、无线电管理等各项事业都取得了可喜的进步。

各位代表，即将过去的2014年，我们凝心聚力，奋发进取，取得了来之不易的成绩。这是党中央、国务院，省委、省政府和市委正确领导的结果，是市人大、市政协以及社会各界大力支持的结果，是全市上下同心同德、奋力拼搏的结果！在此，我代表市人民政府，向全市各族人民，向驻张部队、武警官兵和政法干警，向各民主党派、工商联、人民团体和各界人士，向所有关心支持张家界发展的朋友们，表示衷心的感谢！

各位代表，在总结成绩的同时，我们也要清醒地看到，全市经济社会发展还存在不少问题和不足：一是经济下行压力持续加大，稳增长、促发展面临着不少新的困难和挑战。二是产业规模偏小、结构性矛盾突出，经济发展内生动力严重不足。三是城乡基础设施建设相对滞后，交通、能源、水利、信息、环保等基础条件都还与国内外知名旅游胜地不相适应。四是公共服务设施和体系基础薄弱，教育、卫生、文化、体育等公共服务供给不足、质量不高。五是人民群众生活总体水平仍然较低，部分群众生活还相当困难，扶贫攻坚和民生改善任重道远。六是发展环境不优，项目建设落地慢、落地难的问题仍然存在；在作风持续好转的同时，少数部门和少数工作人员担当精神不强，还存在“门好进、脸好看、事难办”的问题。这些问题，有些是发展进程中的问题，有些是工作上的问题，全市上下必须高度重视，分类施策，坚持不懈加以解决。

二、2015年工作安排

2015年，是全面实施“十二五”规划的收官之年、全面建成小康社会的奋进之年、全面深化改革的关键之年，也是我市加快推进国内外知名旅游胜地建设“1656行动计划”的攻坚之年。全市上下要把握新常态，抢抓新机遇，实现新发展，科学谋划和推进经济社会又好又快发展。

要以改革创新的精神把握新常态。目前，我国经济发展进入新常态，经济增速正从高速增长转向中高速增长，经济发展方式正从规模速度型粗放增长转向质量效率型集约增长，经济结构正从增量扩能为主转向调整存量、做优增量并存的深度调整，经济发展动力正从传统增长点转向新的增长点。认识新常态，适应新常态，引领新常态，是当前和今后一个时期我国经济发展的大逻辑。我们只有把思想和行动统一到中央的认识和判断上来，精准把握新常态，因势利导，扬长避短，奋发有为，才能赢得工作主动权，办好张家界的事情。

要以只争朝夕的劲头抢抓新机遇。新常态必然蕴含新机遇。中央经济工作会议提出了明年经济工作“保持经济稳定增长、培育新的增长点、转变农业发展方式、优化经济发展空间格局、保障和改善民生”五大主要任务。纵观发展大势，我市经济社会发展正面临着诸多新的有利条件：国家继续实施定向调控、结构性调控政策，推进新型工业化、信息化、城镇化、农业现代化同步发展，加快主体功能区和长江经济带战略实施，出台促进旅游业改革发展的若干意见，着力以改善民生实际成效增福祉扩内需，加大武陵山片区区域发展和扶贫攻坚力度，确立湖南“一带一部”发展的新定位，还有省委、省政府不断落实分类指导加快推进全面小康社会建设举措，大力支持大湘西地区经济开发，等等，这些都蕴含着十分难得的新机遇。我们只有紧紧抓住新机遇，才能化压力为动力，不断加快“旅游胜地梦”、“全面小康梦”的实现。

要以攻坚克难的勇气实现新发展。经济新常态在为我们展示新机遇的同时也带来了新挑战。这些挑战，主要是发展进程中存在的困难和问题的挑战，是发展基础、业态水平、要素保障、体制机制、思想观念与新常态不相适应的挑战。在新的一年里，全市上下要直面挑战，担当使命，以奋发有为的姿态迈步新征程，以务实进取的精神争创新业绩，以攻坚克难的勇气实现新发展，奋力夺取旅游提质升级新成效、基础设施建设新突破、城市建设管理新跨越、产业培育发展新进步、人民群众生活新改善、生态环境质量新提升。

各位代表，推进明年经济社会发展，全市上下要以党的十八大和十八届三中、四中全会精神为指导，按照中央和省委经济工作会议、市委六届九次全会的安排部署，紧紧围绕建设国内外知名旅游胜地和全面建成小康社会目标任务，以科学发展观为指导，以改革创新为动力，以提质升级为重点，主动适应经济发展新常态，坚持“稳中求进、好中求快”总基调，落实“三量齐升、分类指导”总要求，实施“提质张家界，打造升级版”总战略，突出基础设施建设，突出产业扩量提质增效，突出城乡统筹发展，突出基本公共服务，突出生态文明建设，增进人民福祉，促进社会和谐，全力推进经济社会平稳健康发展。

——要坚持基础先行、项目支撑。继续开展“项目建设突破年”活动，始终把改善基础条件摆在更加突出的位置，把强化项目建设作为更为关键的举措，致力扩大有效投资、增强发展后劲。

——要坚持旅游带动、产业强市。深入实施“调一进二提三”发展方略，加快培育旅游业为主导、新型工业为支撑、现代服务业为依托、现代农业为基础的具有张家界特色的现代产业体系。

——要坚持以人为本、增进和谐。加强社会建设，坚守工作底线，切实实现好、维护好、发展好人民群众现实利益和长远利益，不断推进基本公共服务均等化，不断满足人民群众对美好生活的新期待。

——要坚持生态优先、绿色发展。强化尊重自然、顺应自然、发展自然的理念，突出生态优势，完善工作机制，以系统工程思路推进生态建设和环境保护，全面打造生态品牌，建设美好绿色家园。

——要坚持改革开放、创新驱动。始终把改革创新、开放合作作为推进经济社会发展的第一动力，着力用改革创新的办法破难题、强保障、稳增长，用开放合作的办法创优势、增活力、促发展。

明年全市经济社会发展的主要预期目标是：生产总值增长9%，其中一、二、三次产业增加值分别增长4%、9%、10%；景点接待旅游人次和旅游总收入分别增长8%和13%，过夜游客增长9%；规模工业增加值增长10%；固定资产投资增长18%；实际利用内、外资分别增长10%和13%；社会消费品零售总额增长12%；进出口总额增长13%；财政总收入增长10%，城镇居民人均可支配收入增长10%，农村居民人均可支配收入增长11%；居民消费价格涨幅和城镇登记失业率分别控制在4%以内，人口自然增长率、安全生产、节能减排实现省定目标；社会事业全面进步，民主法制全面加强，社会大局保持和谐稳定。同时，精心谋划和科学编制好“十三五”发展规划。

实现明年经济社会发展预期目标任务，全市上下要突出抓好九个方面的工作。

（一）保持旅游经济稳定增长，加快旅游转型提质升级

以稳定旅游经济增长为目标，深入推进旅游产品由观光型向观光休闲度假复合型转变，旅游产业由数量型向质量效益型转变，旅游品牌由国内旅游目的地向国内外知名旅游胜地转变，不断提高旅游经济综合竞争力。加快旅游产品产业转型提质升级。着力开发旅游休闲产品，着力提质旅游观光产品。开工建设中心城区南门口、武陵源城区特色街区项目，规范武陵源绿景休闲园、天门洞体育公园二期、万福温泉改造等项目建设，启动澧水半岛和大坪旅游度假区、罗公坪万达文化旅游、张家界水世界等项目建设，完成《魅力湘西》民族文化广场建设工程，加快推进仙人溪特色街区项目前期工作。按照“提质核心、振兴西线、发展东线”的思路，继续抓好武陵源、天门山景区管理和设施改造提质，加速老道湾景区建设，盘活九天洞、茅岩河旅游资源，加快赵家垭、五雷山等景区开发，完成张家界大峡谷玻璃桥二期工程。开工兴建金赛银酒店群和万达、万豪、华邑等酒店项目，加快推进碧桂园、华天城、禾田居高星级酒店二期工程及远大蓝色港湾酒店群建设。加快旅游营销转型提质升级。着力探索和创新适应旅游转型提质升级的营销方式，精心筹办好第四届世界翼装飞行锦标赛、第五届全国自行车挑战赛等重大促销活动，积极利用米兰世博会扩大境外促销，加强与央视等主流媒体和区域营销联盟合作，深化网络、微博、微信等新媒体营销，精心策划事件营销，不断创新政策营销。进一步增强客源地营销针对性，稳定传统客源市场，开发新兴客源市场，搞活银发游、自助游、户外休闲游等特定客源市场。加快旅游服务转型提质升级。提升自驾游服务水平，完善自驾游标识标牌系统，推广使用智慧旅游官网和“安导通”智能服务系统。完成天子山索道改扩建工程，扎实推进游客中心体系建设，加快建设旅游航道和锣鼓塔生态停车场，认真做好张家界旅游客运中心站建设前期准备工作。加快旅游环境转型提质升级。坚持依法治旅，着力推进旅游管理和服务标准化建设。强化“一诚通”管理功能，改善旅游支付环境。采取鼓励措施，加密航班，拓展航线，增开列车，进一步破解旅游大交通“瓶颈”。积极推进景区管理制度创新，切实解决游客排长队问题。大力强化旅游安全管理，切实完善旅游应急指挥系统和旅游联合救援体系。继续抓好“平安满意在张家界”专项活动，进一步提升游客美誉度和满意度。

（二）大力推进产业建设，加快培育现代产业体系

在加快发展现代旅游产业的同时，充分发挥旅游业带动作用，大力构建产业培育发展新格局，推动产业扩量提质增效。加快发展富有比较优势的现代服务业。以信息化为依托，扎实推进服务业做大做强。着力打造现代物流业，加快张家界商贸物流园、汽贸城等项目建设，力争开通航空国际货运业务。创新发展现代金融业，优化金融生态环境，加快组建慈利、桑植农村商业银行，积极发展村镇银行、小额贷款公司、融资性担保公司，力争交通银行来张设立分支机构。精心拓展文化体育业，发挥文化旅游品牌优势，积极招徕承办国际国内重大文化体育交流活动，大力创建新型文化体育服务品牌。改造提升商贸零售业，加快家乐福、中商广场等项目建设，深化农产品流通“新网工程”和直销店建设，兴建农产品批发市场。大力发展电子商务、旅游地产，不断培育新的消费热点。加快发展富有区域特色的新型工业。围绕实施工业产业提质升级“135行动计划”，突出发展生物医药、旅游商品、食品加工、清洁能源、新型材料等特色工业，集中精力在项目开发、规模拓展、特色彰显、品牌创建上下功夫、见成效。扎实推进园区基础设施建设，实施标准厂房、园区道路、污水处理等工程，完成标准厂房年度建设任务，改造好张联路，建成杨林湾路，开工建设阳湖坪污水处理厂。扎实推进园区产业发展，着力推动久瑞生物、智能电力电气设备等项目建设，促进卓达新型建材产业园、旅游商品产业园、康尔佳新上药品生产线等项目落地。全市力争完成工业园区基础设施建设投资4.7亿元，实现新增入园企业20家、工业增加值增长15%的目标。加快发展富有生态品质的现代农业。认真实施两个“百千万”工程，以旅游为依托、市场为导向、生态为品牌，加快转变农业发展方式，落实农业产业提质升级“523行动计划”，提升农业“专、精、特”发展水平。着力推进大鲵品牌发展战略和养殖技术标准规范，新增大鲵养殖规模25万尾。着力扩大蔬菜生产规模和效益，发展商品蔬菜基地4万亩。着力推进优质粮油、水果、烟叶开发生产和茶叶、油茶、花卉苗木、草食牲畜稳定发展。着力开发休闲农业和乡村旅游，拓宽农民增收渠道。

（三）突出重大交通项目建设，加快基础设施建设步伐

围绕强基础、增后劲，坚持“重大项目带动、配套项目跟进”，努力开创基础设施建设新局面。大力实施交通设施项目建设。以打造旅游交通枢纽城市为目标，重点抓好高铁、高速、机场、干线公路“四大交通建设工程”。加快建设黔张常高铁，扎实谋划张吉怀高铁。加快建设张桑高速公路，力争开工建设安慈高速公路，扎实开展宜张、桑龙、张新等高速公路项目前期工作。完成荷花国际机场新航站楼建设并投入使用。开工建设教温、教中、清江、子午西路延伸段等干线公路，加快建设邢大、杨通、零龙、刘洪、官瑞、绕城西线等干线公路和双门岛大桥，完成张沅公路城市段、环景区公路S306段等项目工程。大力实施水利和能源设施项目建设。加快水利基础设施建设，整修病险水库14座，新建村镇供水工程40处，新建和改造泵站13座，新修和加固堤防20公里，疏浚河道15公里。加快水土流失治理项目建设，力争综合治理水土流失面积32平方公里。加快能源基础设施建设，实施永定西、官黎坪、索溪输变电工程，推进慈利、桑植页岩气开发。大力实施通信和信息设施项目建设。加快构建城乡一体化信息网络，积极推广电信网、互联网和有线电视网三网融合。大力推进4G网络建设，新建4G基站900个，完成无线网络、光缆线路年度建设目标任务。健全防灾减灾预报预警信息平台，完成新一代天气雷达系统主体工程建设。

（四）强化城镇建设和管理，加快推进新型城镇化进程

坚持“人本化、特色化、生态化、产业化”发展方向，围绕“幸福城镇、魅力城镇、生态城镇、富裕城镇”建设目标，不断提高新型城镇化发展水平。大力加快中心城市建设步伐，不断完善城市功能。紧扣“拓展新城两区，提质澧水两带”目标，强力实施“四大工程”。实施“道路建设工程”，全面推进“滨水北环线”建设，开工建设南门口滨河路、便河巷滨河路和白马泉高架桥；建成澧兰桥、澧兰中路、桔坪路、木龙滩路；开工重建澧水大桥，兴建沙堤大道，完成天问大道一期工程。实施“棚户区改造工程”，重点推进澧水两岸棚户区改造，加快官黎坪观音大桥东侧、天门山索道以东、边贸市场周边及三角坪棚户区改造建设。实施“绿化精品工程”，围绕创建国家和省级园林城市，加快建设沿水、沿路、沿城、沿景绿化风光带，着力推进澧水风貌带建设取得实质性进展。实施“排污排涝工程”，完成东门溪雨污分流整治工程，启动建设无事溪、仙人溪次干管，进一步完善排污管网。统筹实施县城扩容提质，积极推动旅游风情镇建设。加快推进慈利蒋家坪和桑植老观潭新区建设，有效实施瑞塔铺等5个国家级重点镇、零溪等3个省级示范镇建设，突出抓好以教字垭、天子山、三官寺、洪家关为重点的特色旅游风情镇建设。深入推进城市精细管理，进一步提高游客和市民满意度。在巩固现行“八大整治行动”成果的基础上，强力实施新一轮“八大整治行动”，实现城市管理新跨越。充分发挥街道、社区作用，强化属地管理，全面落实“门前三包”制度。优化征地拆迁工作流程，完善征地拆迁长效机制，加快征地拆迁进程。统筹推进“六城同创”，全面提高城市序化、净化、绿化、亮化、美化水平。完善城乡规划体系，着力塑造城镇个性。编制出台《张家界市新型城镇化发展规划》，形成城乡一体化规划体系。加快城市组团等各项规划编制审批，实现城市控制性详细规划全覆盖。完成镇（乡）域村镇布局规划，启动村庄规划编制。落实规划法定图则管理，严格规范城镇建设秩序，突出抓好对农民建房的规范和服务。坚持“民族传统、时代精神”，建设具有张家界特色的现代建筑，充分运用和提升传统文化、自然山水元素，打造张家界独具魅力的城镇个性。

（五）统筹协调农村发展，加快建设社会主义新农村

按照“城乡一体化”发展思路，着力改善农村生产生活条件，不断增强农村发展活力，加快农村各项事业发展。加快完善农村基础设施。建设高标准基本农田9万亩，开发补充耕地6000亩。完成“百村供电网络改造工程”。建成通畅工程268公里，完成14座渡改桥和危桥改造、299个农村客运站场和招呼点建设任务。修复水毁工程1744处，整修堰塘450口，清淤沟渠190公里，解决好17.38万农村人口饮水安全问题，继续实施抗旱应急水源工程建设。扎实构建农村公共服务体系。强化农业生产经营服务，加快农技、农机、农资、气象、水文、信息等服务网络建设。加强农村文化设施建设，改造完善15个乡镇文化站、152个村文化活动室。不断改善农村上学就医条件，完成11所农村义务教育薄弱学校、10所农村公办幼儿园、16个乡镇卫生院、141个村卫生室的改、扩、新建任务。继续深化农村经济创新发展。积极引导农村土地经营权有序流转，促进多种形式的农业适度规模经营。加快培育农业企业、农村经济合作组织、家庭农场等新型农业经营主体，创新农业经营模式，发展农产品精深加工，搞活农产品流通，切实提高农业产业化和组织化水平。盘活农村集体资产，发展农村集体经济。精准实施扶贫攻坚和新农村建设。坚持以桑植县为主战场，大力推进扶贫攻坚“四基工程”，确保完成省定年度减贫任务。进一步加强基层组织建设，切实提高基层组织保障水平。按照“示范带动、点面结合”的要求，不断深化新农村示范片建设，推动农村全面建成小康社会进程。

（六）多措并举改善民生，加快社会建设全面进步

坚持“守住底线、突出重点、完善制度、引导舆论”的基本思路，更加富有成效地加强社会建设，深化社会治理，推进民生改善。进一步筑牢民生保障安全网。加快就业岗位开发，加强劳动技能培训，统筹抓好城乡就业。加快“五大保险”提质扩面，力争实现应保尽保。继续实施“安居工程”，建设保障性安居工程9694套（户），改造农村危房4000户。建立健全救助机制，加强社会救助平台建设，办好永定区“救急难”和桑植县“医疗救助”国家试点。更加注重“雪中送炭”，保障弱势群体基本生活。提高“五保”集中供养率，新建100所农村幸福院。进一步加快社会事业发展。不断完善教育发展体系，着力抓好义务教育，深入推进义务教育合格学校建设，统筹实施张家界一中等13所中心城区学校改、扩、新建项目；着力普及学前教育，启动第二轮学前教育“三年行动计划”；着力发展现代职业教育，支持张家界旅游职业学校扩建，实施市技工学校二期建设工程；着力提升高等教育，建成张家界航院新校区；着力办好特殊教育，实施特殊教育提升计划。不断完善公共卫生服务体系，全面完成中心城区“三院合一”医疗资源整合，加快推进市人民医院扩建工程；扩大基本药物制度覆盖面，探索实施大病医疗保险；全面落实人口和计生政策，促进人口均衡发展。不断完善公共文化服务体系，建成贺龙体育中心，推进市博物馆配套设施建设，加快市图书馆、图书城建设，开工建设“一宫四中心”（工人文化宫暨职工、老年、青年、妇女儿童四个活动中心），积极争取武陵山博物馆落户张家界；积极开展送戏下乡、送电影下乡，力争实现广播电视“村村通”、“户户响”；切实抓好国家、省级非物质文化遗产项目的申报、保护和传承，启动修复市级馆藏文物和重点文物保护单位。同时，认真办好市第五届运动会和民族乡“逢十庆典”活动，扎实推进国防教育、人防、民兵预备役和“双拥”工作，统筹发展移民、妇女、儿童、老龄、残联、无线电管理等各项社会事业。进一步提升社会综合治理能力。全面落实安全生产责任，深入开展安全生产“打非治违”专项行动，高标准实施食品、药品、农产品“放心工程”，切实加强特种设备等质量安全和校车等学校安全监管，杜绝重特大安全事故发生。深入推进信访制度改革和“三调联动”工作，严格落实信访维稳分级负责制，强化依法治访、领导接访和阳光信访，努力把各种矛盾化解在源头、把群众诉求解决在基层。大力创建“平安张家界”，全面推进反恐处突机制实战化、社区管理网格化和禁毒工作社会化，努力提高社会治理现代化水平。进一步办好惠民实事。市人民政府将在全面落实省定实事项目的同时，扎实办好“在职中小学教师免费体检、农贸市场标准化改造、扶助计生特困家庭、残疾儿童抢救性康复、重性精神疾病患者救治救助”等5件实事。

（七）充分发挥生态环保优势，加快推进生态文明建设

坚持整体推进、重点突破，高标准、高效率抓好生态建设和环境保护。全面创建国家森林城市。按照国家森林城市标准，着力构建森林网络空间格局。大力实施城市绿化、通道绿化、水岸绿化、宜林荒山绿化、镇村绿化、生态恢复、森林资源保护、林产业提质、森林文化建设等九大工程，力争新增人工造林10万亩、封山育林18万亩。切实抓好风景资源和动植物资源保护，全面落实森林防火、森林病虫害防治和地质灾害监测治理。加快推进生态文明示范建设，不断扩大省级以上生态乡镇、市级以上生态村创建面，支持永定区创建省级生态区。深入开展环境整治“六大专项行动”。加快建设水资源管理水质实时监控系统，全面落实水功能区和入河排污口的监督管理，着力实施澧水、溇水流域环境整治工程。依法关闭取缔城区周边采石场、环保炭厂、废旧塑料加工厂、禁养区内养殖场及燃煤锅炉，加强建筑工地夜间施工等噪声整治，搬迁永定城区中心屠宰场。大力开展绿色学校、农庄、小区、酒店和环境友好型企业创建活动，启动武陵源自行车租赁系统建设。深化农村环境综合整治，力争进入全省农村环境综合整治整市推进试点市。积极实施生态主体功能区建设。充分发挥国家主体功能区建设试点示范、全省首批低碳景区试点等平台作用，积极争取和用好用足用活相关政策，不断加快生态市建设进程。加强大鲵资源保护，力争完成张家界大鲵国家级自然保护区功能区调整的相关工作。抓紧实施武陵源核心景区移民搬迁工程，提高世界自然遗产保护水平。加大节能减排工作力度，确保实现省定节能减排目标。

（八）锐意深化改革开放，加快培育和壮大发展动力

深入贯彻落实中央和省委、省政府全面深化改革、坚持扩大开放工作部署，切实加快改革、创新和开放步伐。扎实推进重点领域和关键环节改革。全面完成市、区县两级政府机构改革任务，加快转变政府职能。深入推进旅游综合改革，落实核心景区管理体制改革，实现旅游资源一体化管理，争创首批国家公园。加快工商登记、不动产登记、国有企业、财税体制、金融体制、投融资体制、旅行社公司制改革，着力构建统一开放、竞争有序的市场体系。深化农村综合改革，加快县级公立医院改革，完善食品药品监管体制改革，抓好公车制度改革。积极推进户籍制度改革，着力探索城市公交体制改革，继续推进统计制度改革。统筹推进创新创业。支持鼓励产学研合作创新，突出抓好大鲵、杜仲、茅岩莓等优势资源的研究开发，培育发展高新技术产业。加强自主知识产权保护，鼓励发明专利申请。着力促进大众创业，突出政府引导，强化政策扶持，培育创业主体。营造创新创业浓厚氛围，鼓励外出务工人员回乡创业。深入实施中小企业“百千万”成长工程，力争新增小微企业700家。引导促进非公有制经济发展壮大。全面落实鼓励支持非公有制经济发展各项政策，完善社会服务体系，优化政务服务环境，推动非公有制市场主体创新理念、创新业态、创新技术和管理，壮大实力，加快发展，力争全市非公有制经济增加值增长12%以上。强力推进招商引资。坚持大招商、招大商，力争引进更多战略投资者。强化签约和在谈项目跟踪落实，力争沙堤片区路网等项目尽早正式签约，力争澧水风貌带思善桥特色街区、武陵源文风国际旅游度假区以及大鲵深加工生产基地等项目尽快达成协议。不断扩大开放合作。继续深化口岸开放，开通外国人落地签证业务，增强口岸辐射带动能力，打造武陵山片区对外开放窗口。积极发展对外贸易，加强与沿海产业园区对接与交流，引导外向型企业进驻我市。不断扩大对外合作交流，积极推动国内、国际友城工作可持续发展。

（九）着力转职能改作风，加快建设法治政府

全面加快法治进程，深入改进工作作风，着力构建“职能科学、权责法定、执法严明、公开公正、廉洁高效、守法诚信”的法治政府。加快转变政府职能。依法全面履行政府职能，建立政府“权力清单”，真正落实“法定职责必须为、法无授权不可为”的要求。进一步深化行政审批制度改革，不断简化办事程序。完善政务中心便民服务机制，切实提高行政效能。全面推进依法行政，不断提高政府工作法治化水平。认真落实重大行政决策制度，严格遵循“公众参与、专家咨询、风险评估、合法性审查和集体讨论决定”的法定决策规范。着力加强公务员培训，坚持严格规范公正文明执法，全面落实行政执法责任制。深化普法教育，强化法治举措，坚持以法治凝聚改革共识、规范发展行为、促进矛盾化解、推动经济社会发展。切实加强行政权力制约和监督。按照“公开为常态、不公开为例外”的原则，积极推进行政决策公开、执行公开、管理公开、服务公开、结果公开。主动接受人大及其常委会、人民政协、民主党派和新闻媒体、社会公众等各方面监督，支持审计依法独立行使监督权，让权力在阳光下运行。建立健全转变作风长效机制。充分运用党的群众路线教育实践活动成果，不断强化政府机关和干部队伍改进作风制度建设。大力弘扬勇于担当、敢于担当精神，狠抓制度执行落实，坚决纠正不作为、乱作为，坚决克服懒政、怠政，坚决惩处失职、渎职，形成作风建设新常态。切实履行党风廉政建设主体责任，严格执行党纪政规，坚决抵制各种歪风邪气，全面打造执政为民、风清气正的良好政府形象。

各位代表，时代前行的脚步令人鼓舞，人民寄予的期盼催人奋进。让我们紧密团结在以习近平同志为总书记的党中央周围，在省委、省政府和市委的坚强领导下，同心同德，扎实工作，为加快实现“旅游胜地梦”、“全面小康梦”而努力奋斗！
